# Direct Versus Video Laryngoscopy for Intubating Adult Patients with Gastrointestinal Bleeding

**DOI:** 10.5811/westjem.2015.8.28045

**Published:** 2015-12-01

**Authors:** Jestin N. Carlson, Jason Crofts, Ron M. Walls, Calvin A. Brown

**Affiliations:** * Saint Vincent Hospital, Department of Emergency Medicine, Erie, Pennsylvania; † Brigham and Women’s Hospital, Department of Emergency Medicine, Boston, Massachusetts

## Abstract

**Introduction:**

Video laryngoscopy (VL) has been advocated for several aspects of emergency airway management; however, there are still concerns over its use in select patient populations such as those with large volume hematemesis secondary to gastrointestinal (GI) bleeds. Given the relatively infrequent nature of this disease process, we sought to compare intubation outcomes between VL and traditional direct laryngoscopy (DL) in patients intubated with GI bleeding, using the third iteration of the National Emergency Airway Registry (NEARIII).

**Methods:**

We performed a retrospective analysis of a prospectively collected national database (NEARIII) of intubations performed in United States emergency departments (EDs) from July 1, 2002, through December 31, 2012. All cases where the indication for intubation was “GI bleed” were analyzed. We included patient, provider and intubation characteristics. We compared data between intubation attempts initiated as DL and VL using parametric and non-parametric tests when appropriate.

**Results:**

We identified 325 intubations, 295 DL and 30 VL. DL and VL cases were similar in terms of age, sex, weight, difficult airway predictors, operator specialty (emergency medicine, anesthesia or other) and level of operator training (post-graduate year 1, 2, etc). Proportion of successful first attempts (DL 261/295 (88.5%) vs. VL 28/30 (93.3%) p=0.58) and Cormack-Lehane grade views (p=0.89) were similar between devices. The need for device change was similar between DL [2/295 (0.7%) and VL 1/30 (3.3%); p=0.15].

**Conclusion:**

In this national registry of intubations performed in the ED for patients with GI bleeds, both DL and VL had similar rates of success, glottic views and need to change devices.

## INTRODUCTION

Endotracheal intubation (ETI) is an essential skill in the resuscitation of critically ill patients. ETI ensures oxygenation and ventilation to patients in respiratory distress and helps to protect the airway from gastric contents during regurgitation which may occur during conditions such as gastrointestinal (GI) bleeding. Traditionally, ETI has been performed using direct laryngoscopy (DL) whereby the structures of the airway are directly visualized by the provider, although other techniques have rapidly been adopted by emergency physicians.^1^ Emergency airway management is complicated by many factors including the critically ill nature of the patient population and limited time to prepare for airway maneuvers.^2^ As a result, emergent ETI is associated with increased risk of bradycardia, hypoxia and death.^3^–^5^

Video laryngoscopes (VL) have been developed to help reduce the risk of these complications. VL has been shown to improve ETI success rates and improve the laryngoscopic view.^6^,^7^ VL also improves ETI success across providers.^8^,^9^ Despite these benefits, concerns still arise over the utility of VL in select populations where the camera capturing the image may become obscured by blood or vomitus.^10^ One of these select populations are those patients with upper GI bleeding. Patients with GI bleeding may require ETI for airway protection; however, few data have examined the ideal airway management strategy in this population. We sought to compare intubation outcomes between patients with GI bleeds managed with VL and those managed with DL.

## METHODS

### Study Design

We performed a retrospective analysis of data from a multicenter registry of emergency department (ED) intubations. Each center used in the registry had approval by its institutional review board.

### Setting

NEAR is a collaboration of one community and 12 academic hospitals. Each center had a site investigator who was responsible for ensuring compliance, defined as data entry on >90% of ED intubations, confirmed by comparison of registered patients with computer-generated coding reports for intubation procedures. Contributing center characteristics have been published previously.^11^

Intubation details were recorded onto a standardized intubation form by the intubator, accessed at www.near.edu, using a center-specific login and password. Data were entered using a custom-designed web-based data entry tool and imported directly into a relational database (Microsoft Access®, Microsoft Corporation, Redmond, WA) at the coordinating center (Department of Emergency Medicine, Brigham and Women’s Hospital, Boston, MA). Full details regarding site on-boarding and compliance reporting for NEAR have been previously described.^11^ We collected data on intubations from July 1, 2002, through December 12, 2012.

### Outcomes and Covariates

Variables captured in the database include demographic patient information, indication for intubation, intubation methods, devices used, number of attempts, intubation success or failure, operator characteristics, intubation events and patient disposition. We used operational definitions regarding attempts, methods and adverse events that have been published previously.^11^ An “attempt” was any single effort to place a tracheal tube, which was defined by the leading edge of the laryngoscope blade passing the alveolar ridge. We defined a “method” as any single approach to securing the airway, using specific technique and drugs, such as orotracheal rapid sequence intubation. We report information regarding intubating conditions, intubator discipline and experience, methods, devices used to intubate, and intubation success, stratified by device.

### Selection of Patients

All adult ED patients entered into the database from July 1, 2002 through December 31, 2012, with an attempt at intubation and a medical indication of ‘GI bleed’ were eligible for analysis. Pediatric patients (age<15) were excluded.

### Method of Measurement

We included patient, provider and intubation characteristics. Data were compared between intubation attempts initiated as DL and VL using parametric and non-parametric tests when appropriate. We also evaluated the univariate odds ratios for successful first attempt success for patient, providers and intubation characteristic, including type of laryngoscope (DL or VL). P-values<0.05 were considered to be significant. We completed all analysis using Stata v.12 (Stata Corp, College Station, TX).

## RESULTS

Of the 17,583 adult patients in the NEARIII registry, we identified 325 intubations with the indication listed as “GI bleed.” Of these, 295 had their initial intubation attempted with DL and 30 with VL. DL and VL cases were similar in terms of age, sex, weight, difficult airway predictors, operator specialty (emergency medicine, anesthesia or other) and level of operator training (post-graduate year 1, 2, etc) (Table 1).

First-attempt success was similar between DL and VL (261/295 (88.5%) vs 28/30 (93.3%), p=0.58). Cormack-Lehane views are also similar between the two groups (p=0.78). The need for device change did not differ between DL and VL (2/295 (0.7%) vs 1/30 (3.3%), p=0.15) (Table 2). No matter the initial method, all GI bleed patients identified in the registry were successfully intubated without the need for a supraglottic device or surgical airway.

Type of laryngoscope (DL vs. VL), patient age, height, and weight were not associated with first-attempt success in the univariate analysis (Table 3). Method of intubation (no sedation, sedation only or rapid sequence intubation) did not affect first-attempt success. While increasing number of difficult airway characteristics (DAC) overall did decrease the odds of first-attempt success (OR 0.66 95% CI [0.47–0.94], p=0.02), there was no specific DAC (e.g. neck mobility, Mallampati, or intra-incisor distance) associated with first-attempt success.

## DISCUSSION

We identified 325 intubations in GI bleed patients with 295 where intubation was attempted initially with DL and 30 VL with no difference in intubation outcomes (first-attempt success, glottic view or need to switch device). There were more DL then VL cases. This likely reflects the integration of VL over time within the registry. VL use was rare during the first several years and became more common only near the end of the registry. In the first three years (2002–2004) VL was chosen as the first device in less than 1% of all intubations yet was used in nearly a third of intubations during the last three years of data collection (2010–2012).^1^ It is also possible that DL was preferentially chosen over VL because of the perceived challenges of obtaining a clear image with brisk bleeding.

NEAR includes both community and academic centers. As a result, there are operators of various experience captured within the registry. While the experience of the operators was similar between the VL and DL groups, second-year residents had a lower odds of first-attempt success compared to attendings. The exact reason is likely multifactorial. Previous work has shown that there is a learning curve associated with VL in emergency airway management;^12^ however, this did not occur with first-year residents. First-year residents may be more closely supervised then second-year residents; however, further investigation will be needed to evaluate these differences. Our data confirm previous work showing that overall, trainees perform intubations with a high rate of success.^13^

GI bleeding is a common reason for admission to the hospital with 250,000 to 300,000 admissions in the United States every year and peptic ulcer disease being the most common cause of upper GI bleeds.^14^,^15^ How frequently patients with GI bleed require airway management for airway protection is unknown. In our study using 10 years of multicenter data we identified only 325 intubations. This is a relatively small number given that roughly 30,000 patients die annually in the U.S. as a result of complications from GI bleeds.^15^ While patients with GI bleeds may require airway management in several areas of the hospital (intensive care units, EDs, etc.), we focused our efforts on those that required management in the ED. Given the infrequent nature of ED intubation for GI bleeds, a prospective study comparing DL and VL is unlikely. Our data suggest that patients with GI bleeds requiring airway management in the ED may be managed with VL successfully and with outcomes similar to those managed initially with DL.

Despite these results, concerns may exist that VL will not be of use in this patient population as the camera may become obscured by blood. We found no difference in intubation outcomes between those airways managed initially with DL and VL. While we were unable to quantify the amount of bleeding in these patients, there was no difference in the need to change device (e.g. from VL to DL), suggesting that the phenomenon where the camera may become obscured by blood necessitating device VL abandonment for DL occurs infrequently. VL may in fact be useful in treating these critically ill patients in need of emergent airway protection, especially for operators experienced with VL.

While the use of VL has grown in the ED over the past decade, there are several reasons why providers and hospital may select various devices. There are different types of VL available with different blade shapes and various techniques need for each.^16^ VL is more expensive then traditional DL and may not be as readily available in all facilities.^1^ Some VL offer the advantage of recording ETI attempts to allow for offline review for educational purposes.^17^ Despite these benefits, operators may experience equipment malfunction with VL such as screen failure, although the overall incidence of this is unclear and likely varies by VL device.^18^ Given the low number of VL intubation in the registry, we did not stratify by VL device. Further work may be needed to determine if there are differences in ETI success by the type of VL device used.

## LIMITATIONS

Our study has several limitations. First, this is a self-reported registry and under-reporting of complications, attempts, and adverse events are subject to recall bias. We have no indication that this took place and compliance standards of ≥90% help ensure the population tested is indicative of airway management practices in these centers. We were unable to confirm the amount and location of bleeding. While it is possible that some patients were intubated for shock in the setting of lower GI bleeding, the immediate threat to oxygenation and airway patency is in the setting of brisk upper GI bleeds and it is reasonable to assume these were cases of robust upper GI bleeding. We were also unable to further detail other patient characteristics between the VL and DL groups (anticoagulation use, nasogastric decompression prior to intubation attempts, Child-Pugh score of patients, etc.). The number of DL cases is much higher than VL cases. Previous work has shown that the rate of VL has increased over the time course of NEARIII; however, the majority of airways are still managed with DL in the ED.^1^ While a randomized trial would be the optimal method for addressing this, it is unlikely given the relatively infrequent nature of this disease process even in this national registry spanning nearly a decade of data collection. Finally, the decision to use VL or DL was operator preference. While the DL and VL populations were similar in our measured covariates, it is possible that VL was used by operators who felt more comfortable with their use. It is unknown if similar results would be obtained during random device selection.

## CONCLUSION

In this national registry of ED intubations performed in patients with GI bleeding, DL and VL had similar rates of success, glottic views and need to change devices. VL may be a viable option in this population.

## Figures and Tables

**Table 1:** Patient and operator demographics in a study comparing direct laryngoscopy and video laryngoscopy performed in emergency departments.

Characteristic	Total % (n=325)	DL % (n=295)	VL % (n=30)	P-value
Mean age (SD), years	57.8 (14.9)	57.6 (15.1)	59.9 (13.6)	0.43
Gender, % male (n)	69.2 (225)	69.2 (204)	70 (21)	0.92
Median weight (IQR), kilograms	70 (70–80)	70 (70–80)	70 (65–80)	0.58
DACs				
Median total DACs (IQR)	1 (0–2)	1 (0–2)	1 (0, 2)	0.67
None	28.9 (94)	29.2 (86)	26.7 (8)	0.78
Limited neck mobility (n=315)	5.7 (18)	6.3 (18)	0 (0)	0.16
Limited Mallampati# (n=304)	62.2 (189)	61 (169)	74.1 (20)	0.18
Intra-incisor distance <3 fingers (n=309)	36.3 (112)	36.3 (102)	35.7 (10)	0.95
Thyromental distance <2 fingers	0	0	0	
Obstruction (n=315)	6.4 (20)	7 (20)	0 (0)	0.13
Facial trauma (n=314)	3.8 (12)	4.2 (12)	0 (0)	0.25
Method of intubation				0.34
OTI	4.9 (16)	5.4 (16)	0 (0)	
Sedation only	1.2 (4)	1.4 (4)	0 (0)	
RSI	93.9 (305)	93.2 (275)	100 (30)	
Operator specialty				0.77
Emergency medicine	96.9 (315)	97 (286)	96.7 (29)	
Anesthesia	0.9 (3)	1 (3)	0 (0)	
Other	2.2 (7)	2 (6)	3.3 (1)	
Operator PGY level				0.83
1	10.2 (33)	9.8 (29)	13.3 (4)	
2	34.8 (113)	35.3 (104)	30 (9)	
3	34.8 (113)	34.2 (101)	40 (12)	
4	5.2 (17)	5.1 (15)	6.7 (2)	
Attending	15.1 (49)	15.6 (46)	10 (3)	

*DL,* direct laryngoscopy; *VL,* video laryngoscopy; *SD,* standard deviation; *DAC,* difficult airway characteristics; *IQR,* interquartile range; *OTI,* oral intubation without sedation; *RSI,* rapid sequence intubation; *PGY,* post-graduate year

# Mallampati>1.

**Table 2:** Number of attempts, and Cormack-Lehane grade view by device. All intubations were ultimately successful and none required supraglottic or surgical airways.

Variable	DL %, (n=295)	VL %, (n=30)	p-value
Number of attempts			
1	88.5 (261)	93.3 (28)	0.58
2	8.8 (26)	3.3 (1)	
>2	2.7 (8)	3.3 (1)	
Median number of attempts (IQR)	1 (1-1)	1 (1-1)	0.78
Grade of view			
I	63.6 (185)	65.5 (19)	0.89
II	29.9 (87)	27.6 (8)	
III	5.2 (15)	6.9 (2)	
IV	1.4 (4)	0 (0)	
Device change	0.7 (2)	3.3 (1)	0.15

*DL,* direct laryngoscopy; *VL,* video laryngoscopy; *IQR,* interquartile range

**Table 3:** Univariate odds ratios for first attempt success.

Variable	OR	95% CI	p-value
Video laryngoscope as first device*	1.21	(0.35–4.2)	0.76
Age	1.01	(0.99–1.03)	0.35
Gender**	1.2	(0.58–2.45)	0.63
Weight	1	(0.98–1.01)	0.58
Method			
No medications	(Reference)		
Sedation only	0.14	(0.01–1.67)	0.12
RSI	1.14	(0.25–5.23)	0.87
Operator PGY			
Attending	(Reference)		
1	0.29	(0.07–1.27)	0.1
2	0.27	(0.08–0.95)	0.04
3	0.99	(0.24–3.99)	0.99
4***			
Difficult airway predictors			
Total DACs	0.66	(0.47–0.94)	0.02
Limited neck mobility	0.44	(0.14–1.41)	0.17
Limited Mallampati#	0.69	(0.34–1.47)	0.34
Intra-incisor distance <3 fingers	0.81	(0.4–1.64)	0.56

*OR,* odds ratio; *RSI,* rapid sequence intubation; *PGY,* post-graduate year; *DAC,* difficult airway characteristics

* Reference=Direct Laryngoscopy.

** Reference=Male.

*** All intubations performed by PGY-4 residents were successful on the first attempt.

# Mallampati>1.
